# TPO antibody status prior to first radioactive iodine therapy as a predictive parameter for hypothyroidism in Graves’ disease

**DOI:** 10.1530/ETJ-22-0047

**Published:** 2022-06-10

**Authors:** Sébastien Verdickt, Falco Van Nes, Carolien Moyson, Toon Maes, Paul Van Crombrugge, Annick Van den Bruel, Brigitte Decallonne

**Affiliations:** 1 Department of Endocrinology, University Hospitals of Leuven, Leuven, Belgium; 2 Department of Endocrinology, Imeldaziekenhuis Bonheiden, Bonheiden, Belgium; 3 Department of Endocrinology, OLV Ziekenhuis Aalst-Asse-Ninove, Aalst, Belgium; 4 Department of Endocrinology, AZ Sint Jan Brugge, Brugge, Belgium

**Keywords:** thyroid peroxidase antibodies, Graves’ disease, radioactive iodine therapy, hypothyroidism

## Abstract

**Objective:**

We investigated whether a positive thyroid peroxidase antibody (TPO Ab) status before radioactive iodine (RAI) therapy in patients with Graves’ hyperthyroidism is a predictive factor for developing hypothyroidism post RAI.

**Methods:**

We performed a retrospective study of patients with Graves’ hyperthyroidism with known TPO Ab status, receiving the first administration of RAI. Patients from four thyroid outpatient centres in Belgium receiving their first RAI therapy between the years 2011 and 2019 were studied. Clinical, laboratory, imaging, and treatment data were recorded from medical charts. Hypothyroidism and cure (defined as combined hypo- and euthyroidism) were evaluated in period 1 (≥2 and ≤9 months, closest to 6 months post RAI) and period 2 (>9 months and ≤24 months post RAI, closest to 12 months post RAI).

**Results:**

A total of 152 patients were included of which 105 (69%) were TPO Ab-positive. Compared to TPO Ab-negative patients, TPO Ab-positive patients were younger, had a larger thyroid gland, and had more previous episodes of hyperthyroidism. In period 1, 89% of the TPO Ab-positive group developed hypothyroidism and 72% in the TPO Ab-negative group (*P* = 0.007). In period 2, the observation was similar: 88% vs 72% (*P* = 0.019). In the multivariate logistic regression analysis, a positive TPO Ab status was associated with hypothyroidism in period 2 (adjusted OR: 4.78; 95% CI: 1.27–20.18; *P* = 0.024). In period 1, the aOR was 4.16 (95% CI: 1.0–18.83; *P* = 0.052).

**Conclusion:**

A positive TPO Ab status in patients with Graves’ hyperthyroidism receiving the first administration of RAI is associated with a higher risk of early hypothyroidism.

## Introduction

Radioactive iodine (RAI) has been used for the treatment of patients with Graves’ hyperthyroidism since the 1950s. After a single RAI administration, patients ideally become euthyroid but frequently develop hypothyroidism. On the other hand, hyperthyroidism can persist or relapse necessitating either a second RAI administration or other treatment modalities.

The optimal method for determining the activity of RAI and predicting the thyroid functional outcome remains challenging (1). Several studies identified pre-treatment parameters that could help in the prediction of the thyroid functional outcome such as age, gender, thyroid gland volume, free thyroxine (fT4) and free triiodothyronine (fT3) at diagnosis, and thyroid-stimulating hormone receptor antibody (TSH-R Ab) titer or radiation absorbed dose (2, 3, 4, 5, 6, 7, 8).

Thyroid peroxidase antibodies (TPO Abs) are associated with autoimmune thyroid disease, and their measurement is helpful in the diagnostic workup of hypothyroidism (9). Besides, in euthyroid patients, a positive TPO Ab status is associated with an increased risk for the development of hypothyroidism (10), and patients with silent or post-partum thyroiditis typically have elevated TPO Ab levels (11). In patients with Graves’ hyperthyroidism, TPO Ab status is neither routinely evaluated during the diagnostic workup nor measured to guide management. Treatment with RAI is known to elicit an increase in thyroid antibodies for approximately 1 year, including TPO Ab, considered an immune response secondary to the release of thyroid antigens (12, 13, 14).

To date, the role of the TPO Ab status in patients with hyperthyroidism due to Graves’ disease prior to RAI administration has not been well studied as a predictive factor for thyroid functional outcome.

In this study, we hypothesize that a positive TPO Ab status prior to the first administration of RAI in patients with Graves’ disease increases the incidence of post RAI hypothyroidism.

## Material and methods

### Patient selection and data recorded

**Figure 1:**
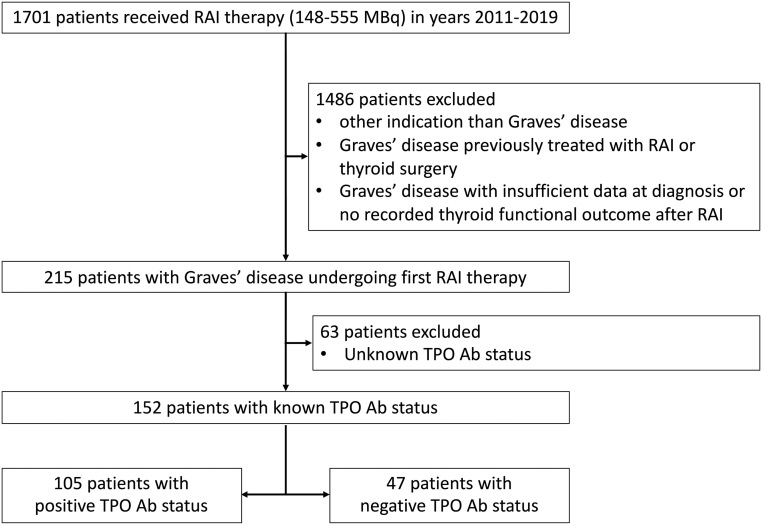
Patient selection.

We performed a retrospective study of medical records from patients with Graves’ disease treated with RAI between 1 January 2011 and 31 December 2019 at four hospitals in Belgium, one academic hospital (University Hospitals Leuven) and three large non-academic hospitals (Imelda Hospital Bonheiden, OLV Hospital Aalst-Asse-Ninove, General Hospital Sint Jan Brugge). The patients were identified by registers of administered RAI kept by the departments of nuclear medicine. The search was limited to patients who were administered RAI in the activity range from 148 to 555 MBq, as lower activities are only used for diagnostic purposes and higher activities for selective ablation post-thyroidectomy in patients with differentiated thyroid cancer. The medical records were screened for eligibility. Only patients with Graves’ disease receiving the first administration of RAI were included. Graves’ disease was defined as a combination of biochemical hyperthyroidism (suppressed TSH with high fT4 and/or fT3) and a positive TSH-R Ab titer or a homogeneous normal to increased technetium (Tc)-99m uptake on thyroid scintigraphy. Patients with a history of thyroid surgery, central hypothyroidism, insufficient data at diagnosis, no recorded thyroid functional outcome after RAI, and patients without TPO Ab assessment prior to RAI were excluded (Fig. 1).

The following data were extracted at the diagnosis of Graves’ disease or at the most recent episode prior to first RAI in case of relapse hyperthyroidism: gender, age, body weight, number of previous episodes of Graves’ hyperthyroidism, history of spontaneous hypothyroidism (in case of female patients), history of post-partum thyroiditis, smoking status, presence of active Graves’ orbitopathy, uptake (%) of technetium on thyroid scintigraphy. Thyroid gland dimensions and presence of nodules >1 cm (maximum diameter) on ultrasonography (US) were recorded based on the US report. The thyroid volume of each lobe was calculated using the formula: width (cm) × length (cm) × depth (cm) × 0.479 for each lobe (15). The thyroid volume was the sum of the volume of both lobes.

The following data were extracted between diagnosis and administration of first RAI: treatment with anti-thyroid drugs (ATD) with or without levothyroxine (L-T4) and treatment with corticosteroids. During and after RAI administration, the following data were collected: administered activity, time of initiation and dosage of L-T4, use of ATDs, execution of thyroid surgery, and second RAI administration.

The following laboratory measurements were recorded at diagnosis: TSH, fT4, TSH-R Abs, TPO Abs. The TPO Ab level closest to the date of diagnosis of Graves’ hyperthyroidism and always prior to RAI administration was recorded, with a limit of 3 months around the diagnosis of last episode of Graves’ hyperthyroidism and before RAI. The TPO Ab status was expressed as positive or negative compared to the manufacturer’s cut-off value. For period 1 (defined as the period between ≥2 and ≤9 months and closest to 6 months post RAI) and period 2 (defined as the period between >9 months and ≤24 months post RAI and closest to 12 months post RAI), TSH and fT4 were recorded. In one centre (University Hospitals Leuven), instead of assessing fT4 directly by immunoassay, total T4 was measured and a free T4 index (FTI) was calculated as total T4 (μg/dL) divided by T3 uptake (%). Therefore, in this study, fT4 is a term encompassing direct measurements of fT4 and calculated FTI. As different laboratory assays for fT4 and TSH-R Abs were used between the centres and during the time period studied (years 2011–2019), results were expressed relative to the upper limit of normal (ULN) and the cut-off value of the assay used, respectively.

The study was approved by the Ethical Committee of the University Hospitals Leuven and the Ethical Committee of each participating centre.

### Definitions of outcomes

Hypothyroidism was defined as a TSH above the ULN, or fT4 below the lower limit of normal, or use of L-T4 excluding patients with concomitant use of ATD as block-replacement therapy.

Cure was defined as the sum of hypothyroidism (as defined above) and euthyroidism, with euthyroidism defined as TSH and fT4 within the normal reference range in a patient not synchronously treated with ATDs or L-T4.

Hyperthyroidism was defined as either not meeting the criteria of cure or having undergone thyroid surgery or having received a second administration of RAI prior to evaluation in period 1 or 2.

### Statistics

The Kolmogorov–Smirnov test was used to verify the assumption of symmetrical distribution of all continuous variables. Symmetrically distributed variables and descriptive data are presented as mean ± s.d., whereas asymmetrical variables are presented as median with first and third quartiles (Q1:Q3). For the analysis of symmetrically distributed variables, unpaired *t*-tests were performed to detect differences between the groups. Non-symmetrically distributed variables were analysed by Mann–Whitney tests. For categorical data, presented as % (*n*), and Pearson’s χ^2^-test was used. In the case of patient numbers ≤5, the χ^2^-test with Yates’s correction for continuity was used.

A multivariate logistic regression was performed with hypothyroidism as a dependent outcome and TPO Ab status as a predictor, adjusted for the following variables: age at diagnosis, fT4 at diagnosis, TSH-R Ab at diagnosis, thyroid volume at diagnosis, ATD preceding RAI (ATD alone or ATD+LT4), and RAI activity. Results are expressed as crude and adjusted odds ratios with 95% CI. A *P* value of <0.05 was considered statistically significant. Analyses were performed with IBM SPSS statistics version 28.0.0 and R Statistical Software (v4.1.3; R Core Team 2021).

## Results

### Baseline patient characteristics

During the study period, out of 1701 patients who received activity of RAI between 148 and 555 MBq, 152 patients with Graves’ hyperthyroidism and a known TPO Ab status were included (Fig. 1). The baseline patient characteristics according to the TPO Ab status are summarized in Table 1. Sixty-nine percent of patients (*n*  = 105) are TPO Ab-positive, and in both groups, most patients are female. Compared to patients with a negative TPO Ab status, patients with a positive TPO Ab status are younger, had more previous episodes of hyperthyroidism, have a higher thyroid gland volume, and more frequently received ATD before administration of RAI.

**Table 1:** Baseline patient characteristics prior to first radioiodine therapy.

	TPO Ab-positive (*n* = 105)	TPO Ab-negative (*n* = 47)	*P* value
Female	77.1% (81)	70.2% (33)	0.362
Age (years)	52.8 ± 13.5	61.3 ± 14.4	**0.001**
Body weight (kg)	69.2 ± 12.4	68.6 ± 12.4	0.768
Active orbitopathy	6.8% (5/74)	7.1% (2/28)	1.000
Active smoker	12.1% (11/91)	5.3% (2/38)	0.394
History of HYPO or PPT	2.9% (3)	4.3% (2)	1.000
First episode of hyperthyroidism	51.4% (54)	76.6% (36)	**0.035**
Laboratory values at diagnosis			
Free T4^a^	1.6 (1.1:2.4)	1.8 (1.3:2.5)	0.293
TSH-R Ab^b^	6.3 (2.7:10.3)	5.4 (2.8:9.0)	0.500
Imaging			
Tc uptake (%)	5.6 (3.6:8.6)	4.4 (2.6:7.5)	0.066
US thyroid volume (mL)	17.3 (12.1:26.6)	13.0 (9.6:21.9)	**0.037**
US ≥ 1 nodule^c^	15.9% (13/82)	32.4% (11/34)	0.081
Therapy preceding RAI			
ATD + L-T4	28.6% (30)	23.4% (11)	0.507
ATD alone	68.6% (72)	63.8% (30)	0.565
CS	0.0% (0)	2.1% (1)	**0.679**
None	2.9% (3)	12.8% (6)	0.043
Interval diagnosis – RAI (days)	77 (46:157)	80 (45:201)	0.951
RAI activity (MBq)	370 (370:518)	370 (296:518)	0.823

Data are presented as % (*n*) for categorical data. Continuous data are presented as mean ± s.d. or median (Q1:Q3) for symmetrically and non-symmetrically distributed variables, respectively. Bold indicates statistical significance, *P* < 0.05.

^a^Values are expressed relatively to the upper limit of normal of the assay; ^b^ Values are expressed relatively to the cut-off of the assay; ^c^Nodules with a maximum diameter of >1 cm on ultrasonography.

ATD, antithyroid drug; CS, corticosteroids; HYPO, spontaneous hypothyroidism; L-T4, levothyroxine; PPT, post-partum thyroiditis; RAI, radioactive iodine; Tc, technetium; US, ultrasonography.

Smoking status and the presence of active Graves’ orbitopathy were recorded in a majority of patients, without significant differences between both groups. The maximum number of prior hyperthyroid episodes was four and this was recorded in two TPO Ab-positive patients. No difference was seen in the degree of thyrotoxicosis and in TSH-R Ab level at diagnosis. In four patients, no TSH-R Ab measurement was available at diagnosis, but they all showed diffusely increased Tc uptake on thyroid scintigraphy.

Imaging was performed in the majority of patients and in similar proportions between both groups. In TPO Ab-positive patients, scintigraphy was performed in 90% and an US in 78% of patients, as compared to 91 and 72% in TPO Ab-negative patients. The time interval between the diagnosis of Graves’ hyperthyroidism and the administration of RAI was similar in both groups. No significant difference in administered RAI activity was noted, with a median activity of 370 MBq in both groups.

### Incidence of hypothyroidism and cure

The time interval between the administration of RAI and the assessment of thyroid function was comparable between the groups, both for period 1 and period 2 (Table 2).

**Table 2:** Thyroid functional outcome in period 1 and period 2.

	TPO Ab-positive	TPO Ab-negative	*P* value
**Period 1**^**a**^	*n* = 103^b^	*n* = 46**c**	
Interval RAI – evaluation (days)	168 ± 40	167 ± 43	0.878
Cure	91% (94)	85% (39)	0.238
Hypothyroidism	89% (92)	72% (33)	**0.007**
Euthyroidism	2% (2)	13% (6)	**0.017**
No cure	9% (9)	15% (7)	0.238
**Period 2**^**d**^	*n***=**94^e^	*n***=**43^f^	
Interval RAI – evaluation (days)	367 (349:414)	366 (345:406)	0.994
Cure	94% (88)	86% (37)	0.146
Hypothyroidism	88% (83)	72% (31)	**0.019**
Euthyroidism	5% (5)	14% (6)	0.084
No cure	6% (6)	14% (6)	0.146

Data are presented as % (*n*) for categorical data. Continuous data are presented as mean ± s.d. or median (Q1:Q3) for symmetrically and non-symmetrically distributed variables respectively. Bold indicates statistical significance, *P* < 0.05.

^a^The thyroid function closest to 6 months after RAI was recorded (with a limit of ≥2 to ≤9 months); ^b^Two patients (*n* = 2/105) have an unknown outcome for period 1; ^c^One patient (*n* = 1/47) has an unknown outcome for period 1; ^d^The thyroid function closest to 12 months after RAI was recorded (with a limit of >9 months to ≤24 months); ^e^Ten patients have an unknown outcome for period 2 (*n*= 10/105), one patient died between RAI and the beginning of period 2 (*n* = 1/105); ^f^Four patients (*n* = 4/47) have an unknown outcome for period 2.

RAI, radioactive iodine.

In period 1, thyroid function was unknown in two TPO Ab-positive patients and in one TPO Ab-negative patient (2% in both groups). In period 2, ten patients were lost to follow-up in the TPO Ab-positive group and four patients in the TPO-negative group (10% vs 9%). One patient with a positive TPO Ab status died between RAI and the start of period 2, due to an unrelated cause.

The incidence of hypothyroidism was significantly higher in the TPO Ab-positive group compared to the TPO Ab-negative group in period 1 (89% vs 72%, *P*  = 0.007) and period 2 (88% vs 72%; *P*  = 0.019) (Table 2).

In case of hypothyroidism, the time interval between RAI and initiation of L-T4 was similar in TPO Ab-positive patients compared to TPO Ab-negative patients (median, 60 days (Q1, 46 : Q3, 102) vs 76 days (Q1, 47 : Q3, 132); *P*  = 0.193). Also, the dose of L-T4 at evaluation in period 1 was similar in both groups (median dose, 100 μg/day (Q1, 75 : Q3, 125); *P*  = 0.592). The same observation was made in period 2 (median, 100 μg/day (Q1, 75 : Q3, 125) vs 87.5 μg/day (Q1, 75 : Q3, 100); *P*  = 0.994).

The incidence of euthyroidism was significantly lower in the TPO Ab-positive group in period 1 (2% vs 13%; *P*  = 0.017) but not in period 2 (5% vs 14%; *P*  = 0.084). As such, the incidence of cure was not different between the groups in period 1 (91% vs 85%; *P*  = 0.238) nor in period 2 (94% vs 86%; *P*  = 0.146).

### Association between a positive TPO Ab status and development of hypothyroidism

In an univariate logistic regression analysis, the OR for hypothyroidism in patients with a positive TPO Ab status was 3.29 (95% CI: 1.35–8.22; *P*  = 0.009) in period 1 and 2.92 (95% CI: 1.17–7.41; *P*  = 0.022) in period 2. In a multivariate logistic regression analysis, adjusting for age at diagnosis, fT4 at diagnosis, TSH-R Ab at diagnosis, thyroid volume at diagnosis, ATD preceding RAI and RAI activity, and the adjusted OR was 4.16 (95% CI: 1.0–18.83; *P*  = 0.052) in period 1 and 4.78 (95% CI: 1.27–20.18; *P*  = 0.024) in period 2 (Table 3). The TPO Ab titre, adjusted for the same variables, was also associated with hypothyroidism in period 2 (Supplementary Table 1, see section on supplementary materials given at the end of this article).

**Table 3:** Logistic regression analysis with positive TPO Ab status as independent variable and hypothyroidism as dependent outcome.

	OR	95% CI	*P* value	Observations
**Period 1**				
Crude	3.29	1.35–8.22	**0.009**	149
Adjusted^a^	4.16	1.00–18.83	0.052	112
**Period 2**				
Crude	2.92	1.17–7.41	**0.022**	137
Adjusted^a^	4.78	1.27–20.18	**0.024**	103

^a^Adjustments for age at diagnosis, fT4 at diagnosis, TSH-R Ab at diagnosis, thyroid volume at diagnosis, ATD preceding RAI, and RAI activity (all continuous variables except the variable ATD preceding RAI). Bold indicates statistical significance, *P* < 0.05.

## Discussion

In this multicentric retrospective study, we investigated if the TPO Ab status in patients with Graves’ disease prior to the first administration of RAI plays a role in the incidence of hypothyroidism and cure, defined as combined hypothyroidism and euthyroidism.

Despite the fact that determination of the TPO Ab titer is currently not required for the diagnosis of Graves’ disease, we observed that it was measured at diagnosis in 71% of patients. This is probably explained by the fact that at the initial workup of a patient with thyrotoxicosis, a positive TPO Ab status with absent TSH-R Ab or low uptake at scintigraphy directs the diagnosis towards silent thyroiditis (11). A positive TPO Ab status was found in 69% of patients which is consistent with other studied cohorts of patients with Graves’ disease (3, 16). In both the TPO-positive and -negative groups, most patients are female. This reflects the higher incidence of Graves’ disease in the female population, and the gender proportion is similar to observations by others (2, 7).

Both TPO Ab-positive and TPO Ab-negative groups had comparable disease activity (hyperthyroxinemia, TSH-R Ab titer, uptake at scintigraphy). Patients with positive TPO Abs were younger, which is in line with the recent observation by Khan *et al.* where higher age was associated with lower odds of having a positive TPO Ab status (17). However, other factors could have interfered since, for example, previous episodes of hyperthyroidism were more frequent in the TPO Ab-positive group, which could have elicited immunization against thyroid antigens, including TPO. The thyroid volume assessed by US, available in 76% of patients, was higher in TPO Ab-positive patients. The high overall incidence of nodularity in both groups is compatible with chronic mild iodine deficiency in Belgium (18).

During the evaluation of thyroid function closest to 6 months post RAI, overall, 89% of patients reached cure, which was similar in both groups and in line with previous observations (7, 19, 20). However, TPO Ab-positive patients were more likely to develop hypothyroidism. This was confirmed in a logistic regression analysis after adjustment for previously established factors associated with thyroid functional outcome after RAI: age, degree of thyrotoxicosis, TSH-R Ab titer, thyroid volume, pre-RAI therapy with ATD, and RAI activity (3, 4, 5, 6, 7, 19, 21, 22).

To our knowledge, there are only two studies that investigated TPO Ab as a predictive parameter for the thyroid functional outcome after RAI (13, 16). In a cohort of 100 patients with Graves’ disease, Catargi *et al.* observed a higher but not statistically different number of patients with a positive TPO Ab status in the hypothyroid group. Recently, Dong *et al.* found that patients with Graves’ hyperthyroidism who developed hypothyroidism 12 months after RAI had significantly increased TPO Ab levels prior to RAI.

Thus, based on our and previous observations, the presence of TPO Ab at diagnosis represents a risk factor for the development of early hypothyroidism after administration of RAI. The underlying mechanism remains elusive. If a positive TPO Ab status would suggest a state of concurrent thyroiditis, a contrary point is that hampered thyroid peroxidase functioning would be expected to decrease RAI organification. It could also be speculated that patients with Graves’ disease and a positive TPO Ab status harbour different intrathyroidal lymphocyte subsets and cytokines interfering with thyrocyte survival in case of a second hit, like radiation (23, 24).

Although the increased incidence of hypothyroidism in the TPO Ab-positive group appears to develop early after RAI, the time of onset of hypothyroidism is comparable in both groups. Half of all included patients were already initiated on L-T4 within 2 months after RAI, highlighting the importance of an early assessment of thyroid function within the first weeks of post RAI. Indeed, the presence of early hypothyroidism after RAI is a risk factor for development or deterioration of orbitopathy, which can be prevented by early administration of L-T4 (25, 26).

This study is limited by its retrospective design, patient number, differences in TPO Ab assays, absence of standardized RAI activity, and absence of data on long-term thyroid functional outcome.

In conclusion, to date, the role of the TPO Ab status in patients with Graves’ hyperthyroidism has not been well studied as a predictive parameter for thyroid functional outcome after first administration of RAI. We show that TPO Ab-positive patients were more likely to develop early hypothyroidism after the first administration of RAI, regardless of previously established factors associated with cure or treatment failure after RAI. The underlying mechanism warrants further investigation. Future studies investigating pre-treatment parameters affecting the outcome after RAI in patients with Graves’ disease should incorporate TPO Ab status as a variable.

## Supplementary Material

Supplementary Table 1. Regression analysis with TPO Ab titre a as independent continuous variable and hypothyroidism as dependent outcome

## Declaration of interest

The authors declare that there is no conflict of interest that could be perceived as prejudicing the impartiality of the research reported.

## Funding

This work did not receive any specific grant from any funding agency in the public, commercial, or not-for-profit sector.

## Author contribution statement

S V, B D: conceptualization, collecting data, statistical analysis, writing of the paper, editing of the paper, final approval of the paper. T M, P V C, A V D B: conceptualization, collecting data, reviewing and editing of the paper, final approval of the paper. C M: statistical analysis. F V N: collecting data.
