# Supplementary material for: TPO antibody status prior to first radioactive iodine therapy as a predictive parameter for hypothyroidism in Graves’ disease
Source: Eur Thyroid J. 2022 Jun 10;11(4):e220047. doi: 10.1530/ETJ-22-0047 (PMC9254270; doi:10.1530/ETJ-22-0047)
Supplement: Supplementary Table 1. Regression analysis with TPO Ab titre a as independent continuous variable and hypothyroidism as dependent outcome [file supplementary_table_1.pdf]

Supplementary Table 1. Regression analysis with TPO Ab titre [a] as independent continuous variable and hypothyroidism as dependent outcome

	OR	95% CI	P value	observations
**Period 1**				
**Crude**	1.33	1.04-1.73	**0.025**	149
**Adjusted [b]**	1.39	0.96-2.07	0.091	112
**Period 2**				
**Crude**	1.37	1.07;1.79	**0.016**	137
**Adjusted [b]**	1.58	1.10;2.37	**0.018**	103

[a] TPO Ab titres were expressed relatively to the cut-off of the assay as different assays were used over time and between hospitals. Due to asymmetrical distribution, transformation was applied (Box-Cox) for normalisation.

[b] adjustments for age at diagnosis, fT4 at diagnosis, TSH-R Ab at diagnosis, thyroid volume at diagnosis, ATD preceding RAI, RAI activity (all continuous variables except the variable ATD preceding RAI)
